# A Parsimonious Ultrasound Radiomics and Ki-67 Model for Estimating MammaPrint Risk Categorization in HR+/HER2− Early Breast Cancer

**DOI:** 10.3390/curroncol33080464

**Published:** 2026-08-04

**Authors:** Yuanjing Gao, Yanwen Luo, Zihan Niu, Mengyuan Zhou, Mengsu Xiao, Tianjiao Chen, Jia Lu, Yuxin Jiang, Bo Pan, Qingli Zhu

**Affiliations:** 1 Department of Ultrasound, Peking Union Medical College Hospital, Chinese Academy of Medical Sciences and Peking Union Medical College, No. 1 Shuaifuyuan, Dongcheng District, Beijing 100730, China; gaoyuanjing@pumch.cn (Y.G.);; 2 Department of Ultrasound, Zhongshan Hospital, Fudan University, Shanghai 200032, China; 3 Department of Breast Surgery, Peking Union Medical College Hospital, Chinese Academy of Medical Sciences, No. 1 Shuaifuyuan, Dongcheng District, Beijing 100730, China

**Keywords:** breast cancer, 70-gene signature (MammaPrint), prognosis, nomogram, radiomics

## Abstract

The 70-gene signature (70-GS; MammaPrint) assay is useful for prognosis assessment in HR+/HER2− early breast cancer, but limited accessibility motivates development of noninvasive alternatives. We retrospectively enrolled 219 women with preoperative grayscale ultrasound and 70-GS results, including a development cohort (*n* = 125), an internal validation cohort (*n* = 53), and a temporally independent validation cohort (*n* = 41). Radiomic features were extracted from manually delineated ROIs using PyRadiomics, and a radiomics score was derived after LASSO selection. Candidate radiomics-only, clinicopathologic-only, and full clinicoradiomic models were explored. To reduce overfitting, we selected a parsimonious model combining the radiomics score and Ki67 as the primary model. The simplified model achieved AUCs of 0.878, 0.816, and 0.831 in the development, internal validation, and temporally independent validation cohorts, respectively. In 1000 bootstrap resamples, the optimism-corrected AUC was 0.872 and the corrected calibration slope was 0.953. Adding the radiomics score to a Ki67-only model significantly improved model fit (likelihood-ratio chi-square = 17.14, df = 1, *p* < 0.001). An ultrasound radiomics and Ki67 model may provide a noninvasive reference for estimating MammaPrint risk categorization, but it should be considered only as a supportive adjunct and not as a replacement for genomic testing.

## Simple Summary

The 70-gene signature (70-GS; MammaPrint) assay helps identify patients with HR+/HER2− early breast cancer who are more or less likely to benefit from chemotherapy, but its cost and limited availability restrict routine use in many settings. In this study, we investigated whether a simplified ultrasound-based model could estimate MammaPrint risk categorization noninvasively. We extracted radiomic features from routine grayscale ultrasound images and combined the derived radiomics score with the Ki67 index. Although larger candidate models were also explored, we selected this parsimonious radiomics and Ki67 model as the primary model because it balanced predictive performance with a lower risk of overfitting. The model showed stable discrimination across the development, internal validation, and temporally independent validation cohorts, and radiomics added significant predictive value beyond Ki67 alone. This approach may provide an accessible adjunctive tool when genomic testing is not readily available.

## 1. Introduction

Breast cancer has emerged as one of the most prevalent cancers and a leading cause of cancer-related death among women [1,2]. Its treatment has undergone a substantial shift from traditional chemotherapy to modern targeted therapies. Genetic profiling plays a crucial role in guiding treatment decisions and assessing patient prognosis [3,4,5]. Tailoring treatment plans based on the patient’s genetic characteristics not only enhances therapeutic efficacy and improves prognosis but also facilitates treatment de-escalation. This personalized approach contributes to better patient tolerance to therapy and alleviates the overall treatment burden. However, Veer LJ’s study [6] showed that the analysis of gene expression can predict the clinical outcomes of breast cancer patients, providing a strategy for selecting those who may benefit from adjuvant therapy. Approximately 60% of breast cancer cases are characterized by the presence of HRs and the absence of HER2, often referred to as HR+/HER2− [7]. Some studies [5,8,9] have shown that a substantial proportion of patients with HR+/HER2− early-stage breast cancer, particularly those with low genomic risk, have favorable outcomes and may safely avoid chemotherapy. 

The 70-GS (MammaPrint) is recommended by the National Comprehensive Cancer Network guidelines for HR+/HER2− early-stage invasive breast cancer patients with node-negative or 1–3 positive lymph nodes to assess the 5- and 10-year distant recurrence risk and guide chemotherapy decisions [10]. The MINDACT trial revealed that 46% of participants were reclassified as genetically low risk by 70-GS, safely avoiding chemotherapy [3]. For elderly patients, the 70-GS offered additional independent prognostic insights into ER-positive, lymph node-negative patients [11]. MammaPrint is a validated genomic assay with prospective outcome-based evidence. Imaging-based models cannot replace genomic testing or directly infer chemotherapy benefit. However, in settings where genomic testing is inaccessible or delayed, an imaging–clinicopathologic model may provide preliminary risk estimation and support discussion regarding whether genomic testing should be prioritized.

Previously, we established a nomogram based on individualized medical history, ultrasound and mammography imaging features and clinicopathological characteristics to predict 70-GS [12], with an AUC of 0.74 in the validation dataset. However, the interpretation of radiographic features is operator dependent, and the range of analyzable signs is relatively restricted. In recent years, radiogenomics has emerged as an important field aiming to bridge imaging phenotypes with underlying molecular and genomic characteristics. Within this framework, radiomics provides a quantitative approach for extracting high-dimensional imaging features that may reflect tumor heterogeneity beyond visual assessment. Radiomics [13,14,15,16,17] can extract high-throughput quantitative imaging features from images, thereby facilitating noninvasive characterization of tumor biology.

In this study, we aimed to develop and validate a parsimonious ultrasound radiomics and Ki67 model to estimate binary (high/low) MammaPrint risk categorization in HR+/HER2− early breast cancer within a Chinese clinical cohort. This study was not designed as a direct comparative diagnostic study against genomic testing; rather, it evaluated whether an ultrasound-based model could estimate assay categorization and provide supportive risk information when genomic testing was unavailable or delayed.

## 2. Materials and Methods

This retrospective study received approval from the Ethics Committee of the Peking Union Medical College Hospital, Chinese Academy of Medical Sciences. Given the retrospective nature of the study and the use of deidentified patient data, the requirement for informed consent was waived by the IRB (Approval Number: K23C2765). The study was conducted in accordance with the ethical standards of the Declaration of Helsinki and its later amendments.

### 2.1. Patient Enrolment

**Figure 1:**
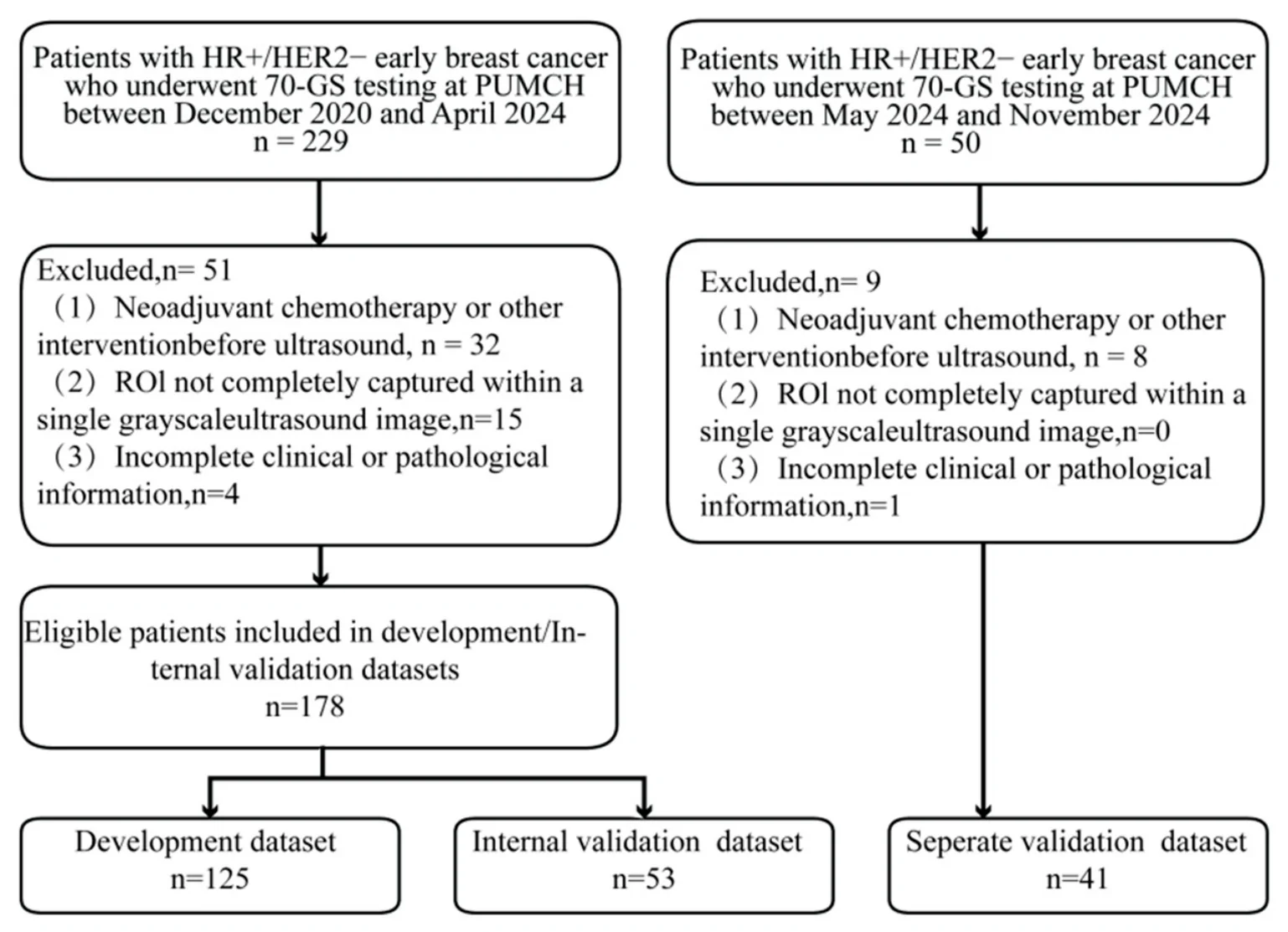
Patient enrollment flow diagram and cohort allocation for model development, internal validation, and temporally independent validation.

Between December 2020 and April 2024, 229 patients with HR+/HER2− early breast cancer received treatment in the Dept. Patients who underwent 70-GS test at Peking Union Medical College Hospital were consecutively included in this study. All patients were numbered, and the computer randomly selected numbers at a ratio of 7:3 to form a development cohort and an internal validation cohort. Between May 2024 and November 2024, 50 patients were enrolled as a temporally independent validation cohort. The 70-GS (MammaPrint) test was performed by ZhenHe Genecast Biotechnology Ltd. (Beijing, China), the sole and exclusive appointed partner of 70-GS in China, by Agendia. The exclusion criteria were as follows: (1) patients who received neoadjuvant chemotherapy or another intervention (biopsy, radiotherapy) before the ultrasound (US) examination; (2) patients whose ROI could not be completely captured within a single greyscale US image; and (3) patients whose clinical and pathological information was incomplete. The 70-gene signature (MammaPrint) risk classification was determined based on a specific scoring range. A score between −0.569 and 0 is classified as high risk, indicating a higher probability of distant metastasis within 10 years. Conversely, a score between 0 and 0.355 is considered low risk, suggesting a lower likelihood of distant metastasis in the same period. Finally, 219 eligible patients with HR+/HER2− early breast cancer who underwent 70-GS testing and had available preoperative grayscale ultrasound images were included. The patient enrollment flow diagram is illustrated in Figure 1.

### 2.2. Data Acquisition

The US features were obtained from the maximum diameter section. The largest diameter of each lesion was recorded on the grayscale US images. Image preprocessing was performed on grayscale US images without considering whether imaging optimization technology, such as harmonics, Sono CT, and XRES, was used. The breast US examinations were performed using four different ultrasound systems (WS80A, Samsung; EPIQ 5/7, Philips; IU22, Philips; and Logiq 9, GE) with linear probes (3–12 MHz, centered at 10 MHz) by qualified radiologists with at least 3 years of experience. Formal post hoc harmonization procedure, such as ComBat, was not applied in the current study. We excluded lesions that could not be completely captured within a single grayscale ultrasound image. It is difficult to delineate the ROI properly if the entire tumor cannot be fully captured within a single image plane. Because the present radiomics workflow was based on 2D grayscale ultrasound images, complete visualization of the lesion on the selected image plane was required for ROI delineation. Lesions larger than 5 cm or lesions that could not be completely captured within a single grayscale ultrasound image were excluded to avoid partial tumor segmentation and non-comparable radiomics feature extraction. In addition, patient age was collected accordingly.

### 2.3. Definition of the ROI

This study was based on 2D grayscale ultrasound radiomics. For each lesion, the analyzed slice was defined as the grayscale ultrasound image showing the maximum tumor diameter, complete lesion visualization, and the clearest visible tumor boundary. A radiologist (Y.G.) with 5 years of breast US experience, who was blinded to the final histopathological diagnosis, drew the ROIs via ImageJ software (version 2.0.2) (National Institutes of Health). To evaluate interobserver variability, another radiologist (Y.L., specializing in breast imaging for 6 years) drew the ROIs in 100 randomly chosen lesions. In addition, to assess intraobserver reliability, Y.G. performed a second delineation of the ROIs from 100 randomly selected images after 1 week via the same procedure. For interobserver and intraobserver reproducibility assessment, ROI delineation was performed on the same preselected image slice for each case. The ROI delineations from the second radiologist and the repeated delineations from the first radiologist were used only for interobserver and intraobserver reproducibility assessment. The primary ROI delineations from the first radiologist were used for subsequent feature extraction and model construction. Discrepancies in ROI delineation were evaluated quantitatively using ICC rather than resolved through manual adjudication or consensus segmentation.

### 2.4. Radiomics Feature Extraction

We applied an open-source python package called PyRadiomics (https://radiomics.io/pyradiomics.html) (accessed on 26 July 2026) to extract radiomic features. Radiomic features were extracted using PyRadiomics version 3.1.0. For transparency, the extraction environment also included SimpleITK 2.2.1 and NumPy 1.24.3. Radiomic features were extracted from the original 2D grayscale ultrasound images and corresponding manually delineated ROIs using PyRadiomics (version 3.1.0) implemented in Python. Only the original image type was enabled, and no additional filters (such as wavelet or Laplacian of Gaussian filters) were applied. Within PyRadiomics, image-intensity normalization was not used (normalize = False), no resampling was performed (resampledPixelSpacing = None), and the default B-spline interpolator was retained. Because the input data were single 2D grayscale ultrasound images, no additional force2D conversion was required. Gray-level discretization was performed using a fixed bin width of 25. The ROI label was set to 255, correctMask was enabled, padDistance was 5, distances was set to [1], and no outlier removal or resegmentation was applied. First-order, shape2D, GLCM, GLRLM, GLSZM, NGTDM, and GLDM features were extracted. Z-score normalization was applied to the extracted radiomic features before downstream feature selection and model construction. The normalization of the extracted features was performed via z score normalization. To ensure a suitable balance between the number of participants and the variables analysed, which is essential for multivariate analysis, we opted for a logistic regression approach enhanced with the LASSO method. Finally, we introduced 102 distinct radiomic features as independent variables.

### 2.5. Model Construction and Performance Evaluation

Before model establishment, we divided the dataset into a development cohort and an internal validation cohort at a 7:3 ratio. The validation data were not used for feature selection or coefficient estimation. Radiomic features were standardized using z-score normalization before model construction to place features on a comparable numerical scale; however, this step should not be interpreted as a substitute for dedicated scanner harmonization. Feature selection was performed in the development cohort using LASSO regression, and the radiomics score was calculated as a linear combination of the selected radiomic features weighted by their corresponding coefficients. The penalty parameter lambda was selected in the development cohort by 10-fold cross-validation using the 1-SE criterion. The radiomics score formula derived in the development cohort was then applied unchanged to the internal validation and temporally independent validation cohorts. We initially explored three candidate logistic regression models: a radiomics model, a clinicopathologic model, and a full clinicoradiomic model. After the initial multivariable analysis, only variables with *p* values < 0.05 were retained for final model construction. Accordingly, the radiomics score and Ki67 index were included in the final simplified model (Ki67-rad model), which was selected as the primary model for the revised analysis. The full clinicoradiomic model and the other candidate models were retained as comparative and sensitivity analyses and are summarized in Supplementary Tables S3 and S4. The internal validation cohort and the temporally independent validation cohort were used only for model evaluation. Model performance was assessed using ROC analysis, AUC, sensitivity, specificity, positive predictive value, negative predictive value, accuracy, F1-score, calibration curves, decision curve analysis, and clinical impact curves. To further assess potential overfitting, bootstrap internal validation with 1000 resamples was performed in the development cohort. Optimism-corrected discrimination and calibration metrics, including the optimism-corrected AUC and calibration slope, were estimated. To evaluate the incremental value of radiomics beyond Ki67, nested likelihood-ratio testing was performed by comparing a Ki67-only model with the Ki67-rad model. Additional scanner-stratified analyses were also performed using recorded ultrasound machine information.

### 2.6. Statistical Analysis

The R (http://www.R-project.org) and EmpowerStats (X&Y Solutions) software programs were used to develop the nomogram. The categorical variables were compared via the chi-square test. For continuous variables, Student’s *t* test was used for comparative analysis of normally distributed data, whereas the nonparametric Mann‒Whitney U test was used to assess the differences between the data that did not meet the criteria for normality. The reported statistical significance levels were all two-sided, and *p* values less than 0.05 were considered statistically significant. An ICC value greater than 0.80 was considered to indicate good reproducibility of radiomic feature extraction with respect to ROI delineation. Nested likelihood-ratio testing was additionally performed to assess the incremental value of the radiomics score beyond Ki67.

## 3. Results

### 3.1. Baseline Characteristics of the Populations

Table 1 shows the baseline characteristics of the study population. This study included a total of 219 patients, with 125 in the development cohort, 53 in the internal validation cohort, and 41 in the temporally independent validation cohort. The 70-GS (MammaPrint) test identified 100 high-risk and 119 low-risk patients. The risk factors, including age, US focality, size, T stage, pathological type, receptor status (estrogen receptor (ER), progesterone receptor (PR), Her-2) and lymph vessel invasion, did not significantly differ between the high-risk and low-risk groups (*p* > 0.05). The radiomics score (*p* < 0.001), lymph node metastatic status (*p* = 0.015), pathological size (*p* = 0.006), histologic grade (*p* < 0.001), and Ki67-positive level (*p* < 0.001) were highly significantly associated with 70-GS risk.

### 3.2. Radiomics Score

**Figure 2:**
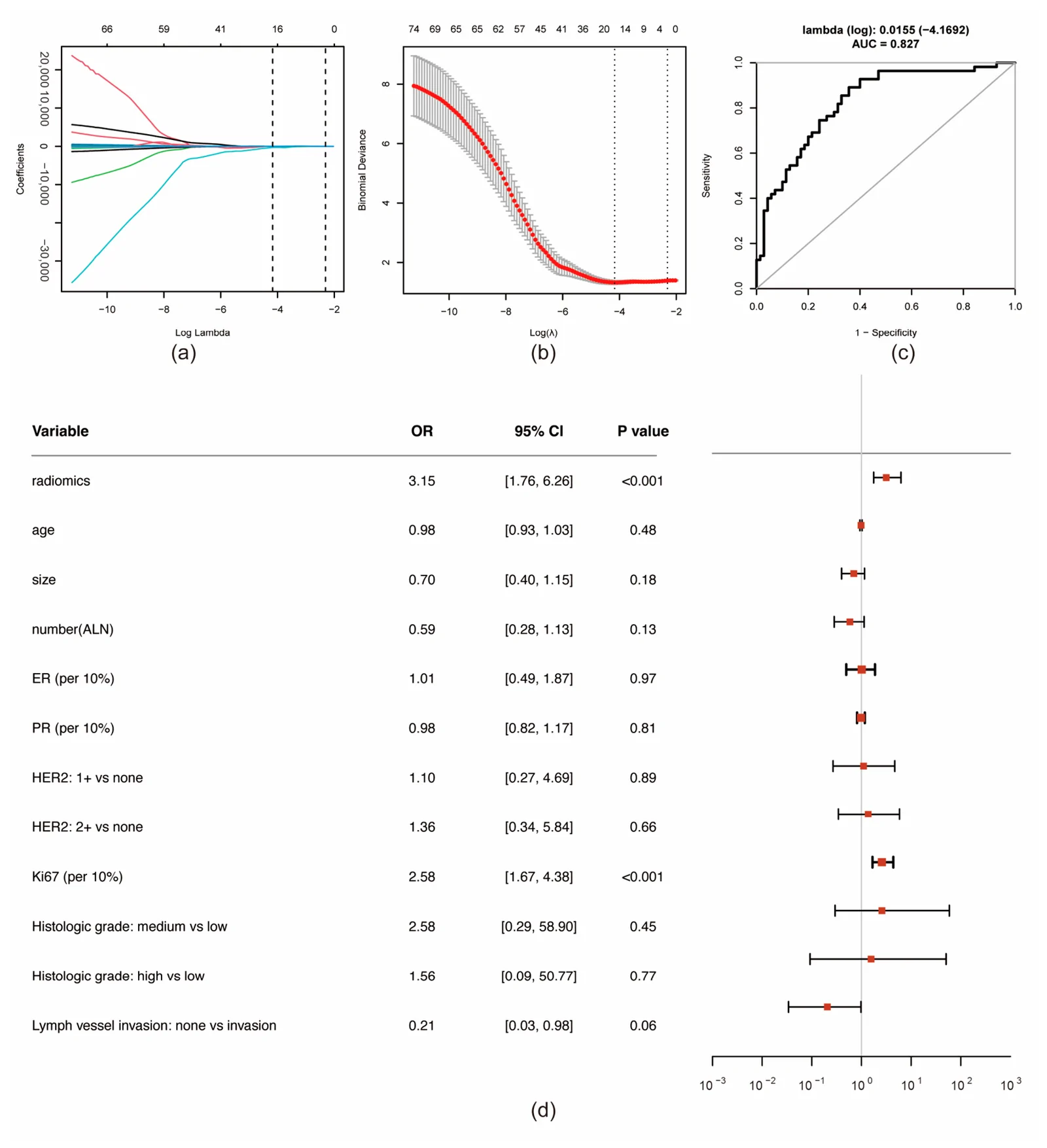
Feature selection and performance evaluation of the LASSO model. (**a**) Tuning parameter λ was selected using 10-fold cross-validation based on the 1 standard error (1-SE) criterion. Dotted vertical lines indicate the optimal values according to the minimum and 1-SE criteria, with the optimal λ determined as 0.0155 [log(λ) = −4.1692]. (**b**) LASSO coefficient profiles of the 102 radiomic features, with a vertical line marking the λ value selected by cross-validation, resulting in 14 nonzero coefficients. (**c**) Receiver operating characteristic (ROC) curve of the radiomics score for predicting high-risk categorization of the 70-gene signature test after LASSO regression. (**d**) Forest plot of multivariate logistic regression analysis showing the included risk factors. OR, odds ratio. In panel (**d**), ORs were derived from multivariable logistic regression and represent the association between each variable and high-risk 70-GS categorization. ER,PR,Ki67 was reported per 10% increase.

In the development cohort, 102 radiomic features were reduced to 14 potential predictors via the LASSO regression model (Figure 2a,b). Table 2 lists the variable names that constitute the radiomic score formula, along with their corresponding coefficients. Additionally, it includes the Spearman correlation coefficients between each variable and the outcome. The radiomics score was a model-derived composite index calculated as a weighted linear combination of the selected standardized radiomic features; therefore, its absolute numerical scale was model-dependent and should not be interpreted as a directly biologically meaningful unit. Model discrimination depended on between-patient differences rather than on the absolute magnitude of the score. The radiomic signature showed discriminatory ability in the development cohort (Figure 2c).

### 3.3. Feature Selection

From the multivariate analysis forest plot (Figure 2d), only the radiomics score and Ki67 index were statistically significant predictors of high-risk 70-GS (both *p* < 0.05). Other variables, including age, tumor size, number of lymph nodes, ER, PR, Her2 status, histologic grade, and lymph vessel invasion, were not significant (*p* > 0.05). To reduce overfitting and improve model parsimony, we prespecified a final simplified model using only these two predictors. The reproducibility of radiomic feature extraction with respect to ROI delineation was acceptable, with intraobserver ICCs ranging from 0.764 to 0.999 and interobserver ICCs ranging from 0.794 to 0.996. Detailed ICC results and the radiomic features excluded because of ICC < 0.80 are provided in Supplementary Table S1.

### 3.4. Establishment and Validation of Nomogram Models

**Figure 3:**
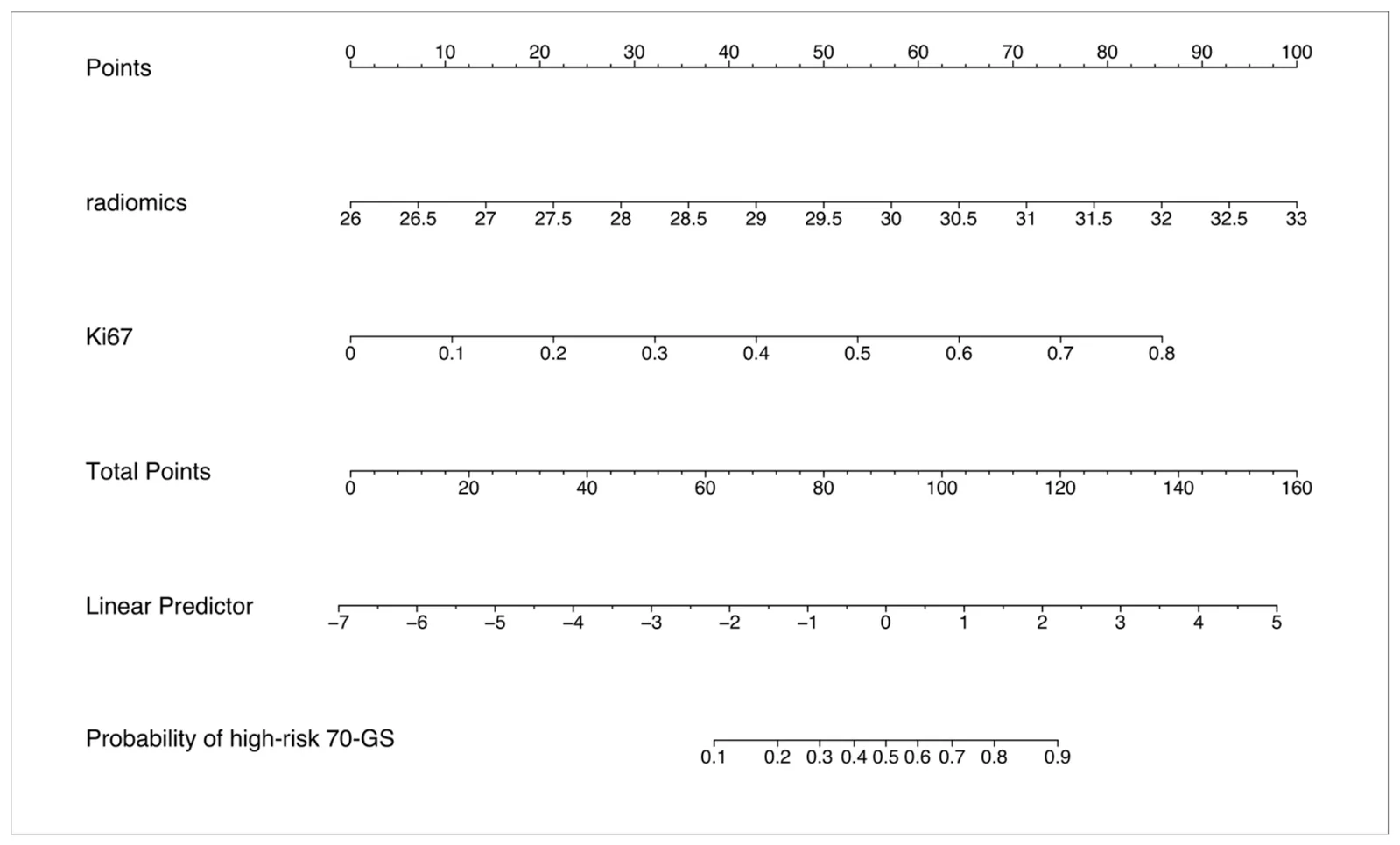
Nomogram of the simplified radiomics and Ki67 model for predicting binary MammaPrint risk categorization: The nomogram was constructed using the radiomics score and Ki67 index in the development cohort. For each predictor, a vertical line can be drawn upward to the points axis to determine the corresponding score. The total points are then projected to the probability scale to estimate the likelihood of high-risk 70-GS/MammaPrint categorization.

We selected a parsimonious Ki67-radiomics model as the final model for the revised analysis. A nomogram based on these two predictors was then constructed to estimate the probability of a high-risk 70-GS result (Figure 3), and the corresponding regression coefficients are provided in Supplementary Table S2. The nomogram should be regarded primarily as a visual representation of the underlying regression model rather than as a manual substitute for genomic testing, and probability estimation would be more suitably implemented through the regression equation or an electronic calculator in practice. For comparative purposes, a clinical model (age, number of lymph nodes, ER status, PR status, Her-2 status, Ki67 index, histological grade, and lymph vessel invasion), a radiomics–clinicopathologic model (combining all the features mentioned above), a Ki-67-only model, and a radiomics model were also evaluated as secondary models.

**Figure 4:**
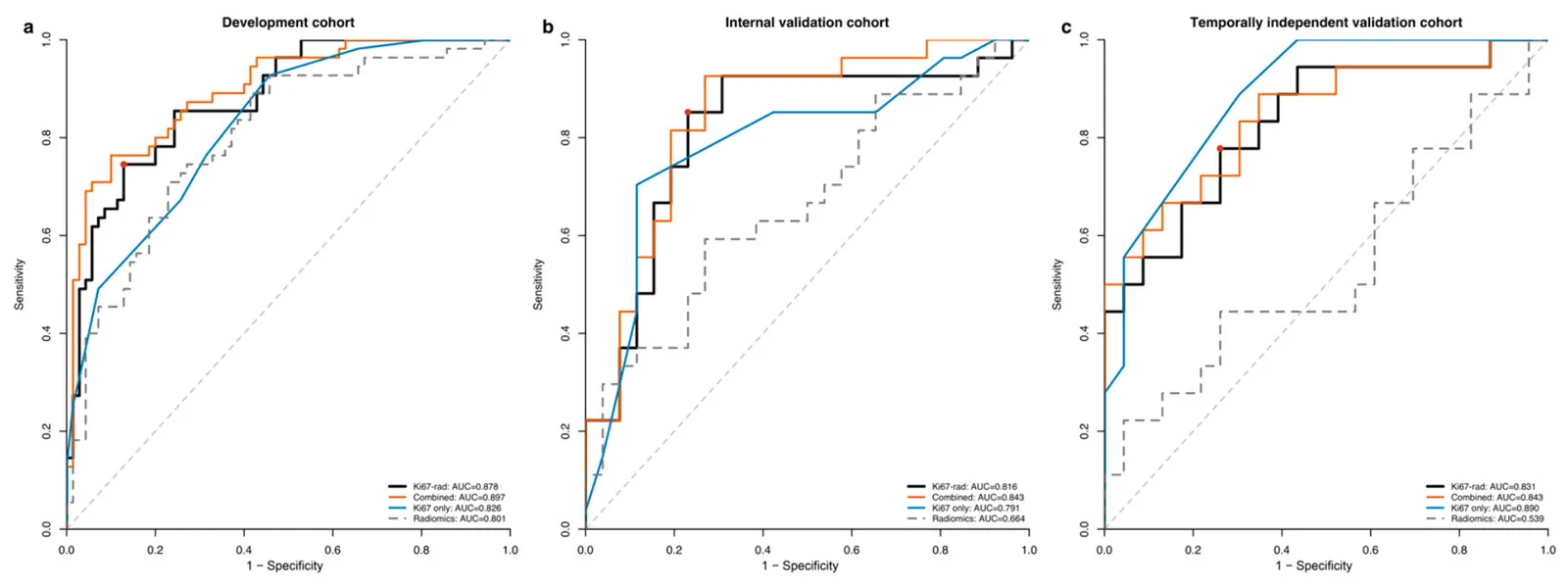
Receiver operating characteristic (ROC) curves of the simplified Ki67-radiomics model and comparative candidate models for predicting high-risk 70-GS/MammaPrint categorization in the development cohort (**a**), internal validation cohort (**b**), and temporally independent validation cohort (**c**). Comparative models included the combined clinicoradiomic model, clinicopathological model, Ki67-only model, and radiomics model. AUC, area under the receiver operating characteristic curve.

The Ki67-radiomics model showed good discriminative ability, with AUCs of 0.878 (95% CI, 0.819–0.936) in the development cohort, 0.816 (95% CI, 0.692–0.940) in the internal validation cohort, and 0.831 (95% CI, 0.702–0.960) in the temporally independent validation cohort (Figure 4). In paired DeLong tests, the Ki67-radiomics model was not significantly different from the combined model in any cohort (development cohort: *p* = 0.251; internal validation cohort: *p* = 0.379; temporally independent validation cohort: *p* = 0.801). Compared with the radiomics model, the Ki67-radiomics model achieved significantly higher AUCs in all three cohorts (*p* = 0.010, 0.028, and <0.001, respectively). Compared with the Ki-67-only model, the Ki67-radiomics model showed a significant improvement only in the development cohort (*p* = 0.041), whereas the differences were not significant in the internal validation cohort (*p* = 0.523) or the temporally independent validation cohort (*p* = 0.299). Detailed diagnostic performance metrics are provided in Supplementary Table S3, and paired DeLong comparisons are provided in Supplementary Table S4.

### 3.5. Model Performance Evaluation

The predictive performance of the Ki67-rad model was further evaluated through bootstrap internal validation and supplementary sensitivity analyses. In the development cohort, the apparent AUC of the Ki67-rad model was 0.878, whereas the optimism-corrected AUC was 0.872. The optimism-corrected calibration slope was 0.953, indicating limited optimism and favorable internal calibration stability. To evaluate the incremental contribution of radiomics, we compared a Ki67-only model with the Ki67-rad model. In nested likelihood-ratio analysis, adding the radiomics score significantly improved model fit (LR chi-square = 17.14, df = 1, *p* < 0.001). In the scanner-stratified analysis, the radiomics–clinicopathologic model achieved an AUC of 0.840 (95% CI, 0.741–0.939) in the iu22 group and 0.869 (95% CI, 0.810–0.927) in the EPIQ group. There was no significant difference in AUC between these two principal machine groups (*p* = 0.622). Bootstrap internal validation and incremental value results for the Ki67-rad and Ki67-only models are summarized in Supplementary Table S5, and comparative performance metrics for the full clinicoradiomic model are provided in Supplementary Tables S3 and S4.

### 3.6. Model Assessment Through Calibration, Cost‒Benefit, and Clinical Impact

**Figure 5:**
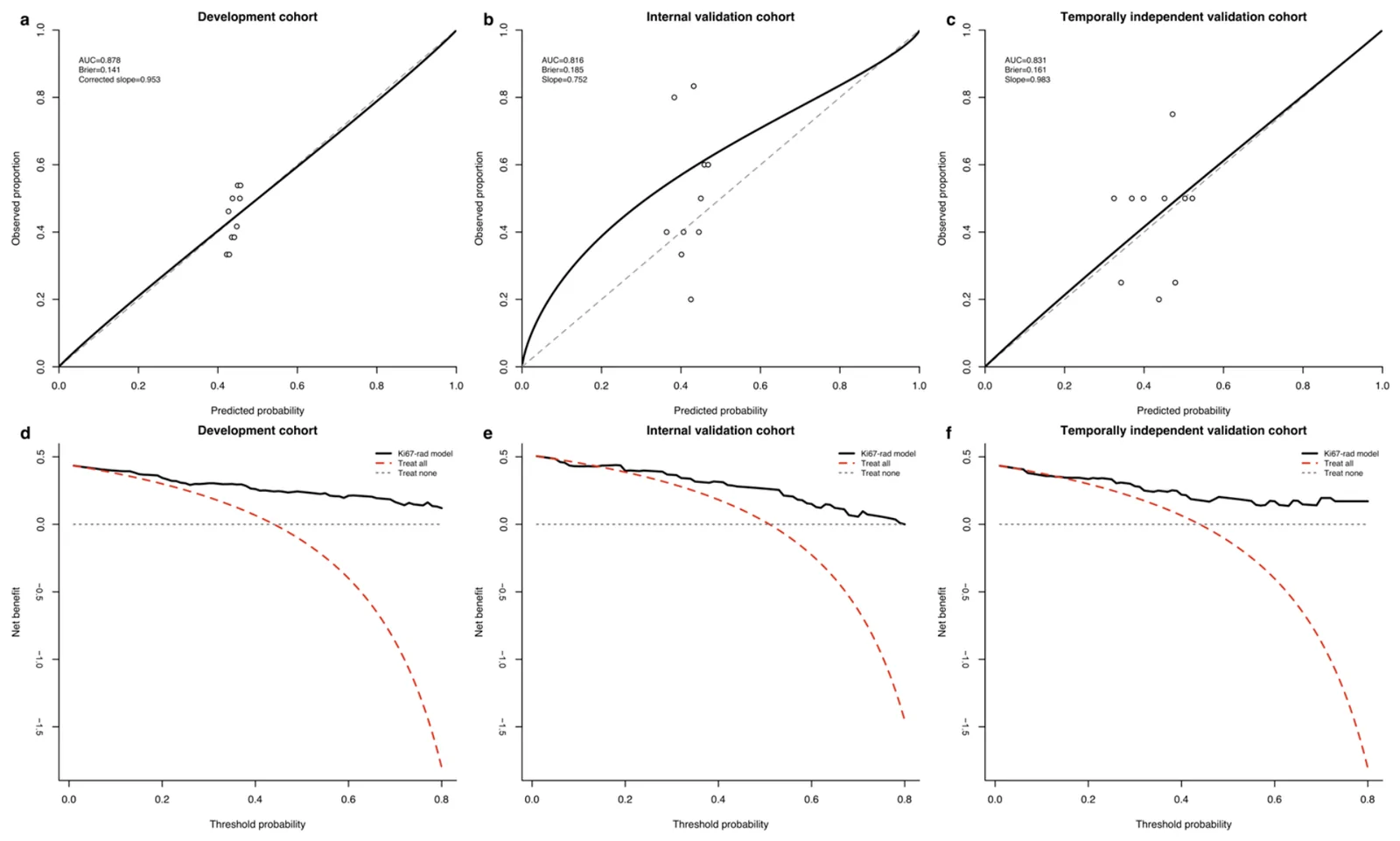
Calibration plots and decision curve analysis of the Ki67-radiomics model. Calibration plots in the development cohort (**a**), internal validation cohort (**b**), and temporally independent validation cohort (**c**). Decision curve analysis in the development cohort (**d**), internal validation cohort (**e**), and temporally independent validation cohort (**f**). The dashed gray line indicates ideal calibration; the solid black line indicates the model curve. In the decision curve analysis panels, the solid black line represents the Ki67-radiomics model, the red dashed line represents the treat-all strategy, and the gray dotted line represents the treat-none strategy.

The calibration plots for the Ki67-rad model in the development, internal validation, and temporally independent validation cohorts demonstrated acceptable agreement between predicted probabilities and observed outcomes (Figure 5a–c). Overall, the close alignment of the bias-corrected curves with the ideal reference line indicates that the Ki67-rad model provided reasonably accurate probability estimates across cohorts. Decision curve analysis further suggested that the Ki67-rad model yielded potential clinical net benefit across clinically relevant threshold probabilities (Figure 5d–f).

**Figure 6:**
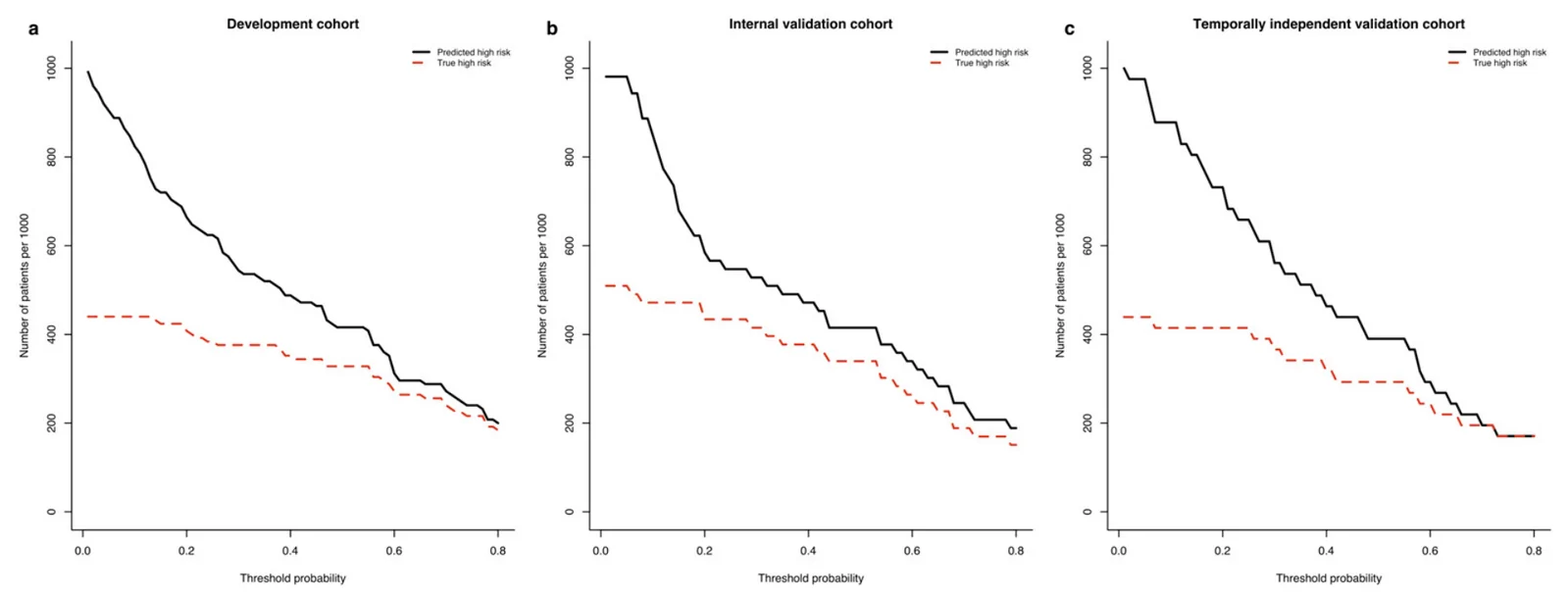
Clinical impact curves of the Ki67-radiomics model in the development cohort (**a**), internal validation cohort (**b**), and temporally independent validation cohort (**c**). The solid black line represents the number of patients classified as high risk by the model at each threshold probability, and the red dashed line represents the number of true high-risk patients.

The clinical impact curves (Figure 6a–c) illustrate the practical implications of the Ki67-rad model. The predicted high-risk curve showed reasonable agreement with the observed MammaPrint high-risk status across a range of threshold probabilities, supporting the potential clinical applicability of the Ki67-rad model as an adjunctive risk-stratification tool.

## 4. Discussion

In this study, we developed and validated a parsimonious ultrasound-based nomogram combining the radiomics score and Ki67 for estimating binary 70-GS/MammaPrint risk categorization in HR+/HER2− early breast cancer. The simplified model showed stable discrimination across the development, internal validation, and temporally independent validation cohorts, with AUCs of 0.878, 0.816, and 0.831, respectively. The present model should be interpreted as an exploratory adjunct for estimating 70-GS risk categorization, not as a substitute for the MammaPrint assay. Because no recurrence-free survival, distant metastasis-free survival, overall survival, or chemotherapy benefit endpoints were available, the model cannot be assumed to provide prognostic or predictive information equivalent to genomic testing.

To our knowledge, this is among the first studies in a Chinese cohort to prioritize a parsimonious ultrasound radiomics and Ki67 model for estimating binary MammaPrint risk categorization [12]. Many studies [18,19] have shown that radiomics can capture quantitative imaging information beyond visual assessment and may provide imaging biomarkers related to tumor biology. In our revised analysis, we initially explored broader clinicopathologic and clinicoradiomic candidate models but ultimately prioritized the simplified model because it provided a more favorable balance between discrimination, interpretability, and robustness to overfitting. Compared with more complex models, a two-predictor model may also be easier to interpret and potentially easier to implement in routine clinical practice.

Regarding ROI segmentation, our study used 2D maximum-diameter sections rather than 3D volumetric segmentation. Although 2D analysis may miss heterogeneity outside the selected image plane, it remains clinically relevant because it aligns with routine ultrasound practice and is more feasible for near-term translation. In addition, the independent delineations from the second radiologist and the repeated delineations from the first radiologist served as a reproducibility assessment, allowing us to retain only radiomic features with acceptable stability (ICC > 0.80) for model construction. Nevertheless, because the final model was developed from the primary delineations of the first observer, residual observer dependence in the resulting radiomics score cannot be fully excluded.

From a clinical perspective, the radiomics score and Ki67 may reflect complementary aspects of tumor biology. The radiomics score captures quantitative information related to lesion shape, texture, and intratumoral heterogeneity on ultrasound, whereas Ki67 reflects proliferative activity at the pathological level. Imaging phenotypes such as lesion shape irregularity, echotexture complexity, and intratumoral heterogeneity may indirectly reflect downstream manifestations of tumor biology, including cellular density, stromal remodeling, and proliferative activity. Although individual ultrasound radiomic features cannot be directly mapped to specific genes within the MammaPrint panel, combining the radiomics score with Ki67 may still capture complementary imaging-based and biologic information, which may help explain the stable discrimination observed across cohorts. The selected radiomic features should not be interpreted as individually validated biological markers, because their mechanistic relationship with MammaPrint categorization remains uncertain. In addition, the Spearman correlation coefficients shown for individual features were descriptive only; feature selection was based on their joint contribution within the LASSO-derived multivariable radiomics signature rather than on the magnitude of their univariable correlations alone. Compared with more complex deep learning approaches, this simplified nomogram also offers greater transparency and may facilitate clinical interpretation and communication. Nevertheless, we acknowledge that deep learning and explainable AI approaches may further improve performance and feature representation in future larger multicenter studies [20,21,22,23,24].

Multigene sequencing enables the evaluation of recurrence risk in patients with early-stage breast cancer, thereby indicating the need for adjuvant chemotherapy. This strategy not only enhances treatment efficacy but also minimizes unnecessary treatment-associated adverse effects [25]. However, for Chinese patients, the cost of gene sequencing reaches 50–75% of the average annual income of Chinese residents [26]. The high cost means that many patients cannot afford to benefit from this test.

Numerous studies developing predictive models for diverse prognostic indicators have identified key prognostic factors, including clinicopathological features such as age, tumor size, percentage of ER- and PR-positive cells, and the Ki-67 index, which are strongly correlated with patients’ clinical outcomes [27,28,29]. A key finding is that radiomics provided significant incremental value beyond Ki67 alone. In nested likelihood-ratio analysis, adding the radiomics score to a Ki67-only model significantly improved model fit. This result supports the hypothesis that ultrasound-derived radiomic information captures complementary aspects of tumor phenotype that are not fully reflected by Ki67 alone. Although larger multivariable models showed slightly higher apparent discrimination, they were also more vulnerable to overfitting, which motivated our decision to prioritize the parsimonious model.

Our research has several limitations. First, its retrospective design may have introduced selection bias and other unavoidable inconsistencies. Second, the internal validation cohort (*n* = 53) and temporally independent validation cohort (*n* = 41) were relatively small, leading to wide confidence intervals. In addition, between-cohort differences in some clinicopathologic variables may have contributed to fluctuations in discrimination and calibration estimates. Notably, ER and PR category distributions differed across cohorts; given the moderate sample size and the temporal definition of the independent validation cohort, such imbalances were not unexpected, but they may still have affected model performance and calibration. Nevertheless, the AUC point estimates of the simplified model were reasonably consistent across the development, internal validation, and temporally independent validation cohorts, and bootstrap internal validation yielded an optimism-corrected AUC of 0.872 and a calibration slope of 0.953, supporting the internal stability of the model. Although the temporally independent validation cohort provided an additional test of temporal robustness, it originated from the same institution and therefore should not be interpreted as true external multicenter validation. Third, ultrasound images were acquired using multiple scanner platforms. Because formal harmonization was not performed across the four ultrasound systems, residual scanner-dependent bias may remain despite the use of a uniform feature-extraction pipeline, ICC-based feature filtering, and supplementary scanner-stratified analysis. In retrospective 2D grayscale ultrasound radiomics, dedicated harmonization remains methodologically challenging because ultrasound image appearance is strongly affected by vendor-specific processing and acquisition-dependent settings. Although supplementary scanner-stratified analyses suggested no major performance difference between the two principal ultrasound systems, scanner-related variability in radiomic features remains an important issue for future multicenter validation. Prospective multicenter studies with standardized imaging protocols, scanner-balanced cohorts, feature harmonization, and external validation are still needed. Fourthly, 2D radiomics may miss heterogeneity outside the selected imaging plane. Finally, because the study included only patients who underwent MammaPrint testing, the cohort represents a clinically selected subgroup with potential treatment-decision uncertainty rather than the full HR+/HER2− early breast cancer population. Therefore, the generalizability of the model to unselected patients remains limited.

## 5. Conclusions

In conclusion, this study developed a parsimonious ultrasound radiomics and Ki67 nomogram for estimating binary 70-GS/MammaPrint risk categorization in HR+/HER2− early breast cancer. The model showed stable discrimination across the development, internal validation, and temporally independent validation cohorts, demonstrated limited optimism in bootstrap internal validation, and provided significant incremental value beyond Ki67 alone. It may serve as a noninvasive adjunctive tool when genomic testing is not readily available, although prospective multicenter validation with clinical outcome endpoints remains necessary before routine implementation.

## Figures and Tables

**Table 1:** Comparison of clinicopathological characteristics and imaging features between datasets.

Dataset	Overall N = 219	Development Dataset N = 125	Internal Validation Dataset N = 53	Temporally Independent Validation Dataset N = 41	*p* Value
**Age (mean ± SD)**	50.16 ± 11.03	50.02 ± 11.25	50.53 ± 10.54	50.07 ± 11.19	0.961
**Age group**					0.143
<40	40 (18.3)	18 (14.4)	12 (22.6)	10 (24.4)	
40~49	71 (32.4)	48 (38.4)	12 (22.6)	11 (26.8)	
50~59	62 (28.3)	35 (28.0)	19 (35.8)	8 (19.5)	
≥60	46 (21.0)	24 (19.2)	10 (18.9)	12 (29.3)	
**US focality**					0.111
Unifocal	186 (84.9)	111 (88.8)	44 (83.0)	31 (75.6)	
Multifocal	33 (15.1)	14 (11.2)	9 (17.0)	10 (24.4)	
**Tumor Size (maximum diameter of tumor) (median [IQR])**	1.90 [1.40, 2.60]	2.00 [1.40, 2.60]	2.00 [1.30, 2.70]	1.60 [1.40, 2.00]	0.143
**Radiomics Score (median [IQR])**	30.00 [29.33, 30.66]	30.21 [29.31, 30.69]	29.93 [29.43, 30.72]	29.95 [29.53, 30.44]	0.886
**pT**					0.056
T1	130 (59.4)	68 (54.4)	31 (58.5)	31 (75.6)	
T2	84 (40.6)	52 (45.6)	22 (41.5)	10 (24.4)	
**LNM**					0.534
No metastasis	110 (50.2)	58 (46.4)	29 (54.7)	23 (56.1)	
1–2 LN metastasis	105 (47.9)	63 (50.4)	24 (45.3)	18 (43.9)	
≥3 LN metastasis	4 (1.8)	4 (3.2)	0 (0.0)	0 (0.0)	
**Histological grade**					0.370
G1	28 (12.8)	12 (9.6)	8 (15.1)	8 (19.5)	
G2	173 (79.0)	100 (80.0)	42 (79.2)	31 (75.6)	
G3	18 (8.2)	13 (10.4)	3 (5.7)	2 (4.9)	
**Pathological type**					0.400
Invasive ductal carcinoma	206 (94.1)	118 (94.4)	51 (96.2)	37 (90.2)	
Invasive lobular carcinoma	10 (4.6)	6 (4.8)	2 (3.8)	2 (4.9)	
Others	3 (1.4)	1 (0.8)	0 (0.0)	2 (4.9)	
**ER positivity (Median [IQR])**	0.90 [0.82, 0.93]	0.90 [0.80, 0.90]	0.90 [0.90, 0.95]	0.90 [0.80, 0.90]	0.267
**ER positive level**					<0.001
Negative (0)	1 (0.5)	1 (0.8)	0 (0.0)	0 (0.0)	
Mild (1+)	1 (0.5)	0 (0.0)	0 (0.0)	1 (2.4)	
Moderate (2+)	28 (12.8)	9 (7.2)	3 (5.7)	16 (39.0)	
Strong (3+)	189 (86.3)	115 (92.0)	50 (94.3)	24 (58.5)	
**PR positivity (Median [IQR])**	0.80 [0.40, 0.90]	0.80 [0.40, 0.90]	0.80 [0.50, 0.90]	0.70 [0.30, 0.90]	0.321
**PR positive level**					<0.001
Negative (0)	23 (10.5)	16 (12.8)	5 (9.4)	2 (4.9)	
Mild (1+)	55 (25.1)	35 (28.0)	17 (32.1)	3 (7.3)	
Moderate (2+)	31 (14.2)	9 (7.2)	1 (1.9)	21 (51.2)	
Strong (3+)	110 (50.2)	65 (52.0)	30 (56.6)	15 (36.6)	
**HER2**					0.141
-	44 (20.1)	28 (22.4)	12 (22.6)	4 (9.8)	
1+	92 (42.0)	45 (36.0)	24 (45.3)	23 (56.1)	
2+/ISH-negative	83 (37.9)	52 (41.6)	17 (32.1)	14 (34.1)	
**Ki67 (median [IQR])**	0.25 [0.15, 0.40]	0.25 [0.15, 0.40]	0.20 [0.15, 0.40]	0.30 [0.15, 0.40]	0.897
**Ki 67 positive level**					0.491
Low (<20%)	79 (36.1)	42 (33.6)	19 (35.8)	18 (43.9)	
High (≥20%)	140 (63.9)	83 (66.4)	34 (64.2)	23 (56.1)	
**Lymph vessel invasion**					0.381
No	190 (86.8)	105 (84.0)	48 (90.6)	37 (90.2)	
Yes	29 (13.2)	20 (16.0)	5 (9.4)	4 (9.8)	
**MammaPrint**					0.675
Low risk	119 (54.3)	70 (56.0)	26 (49.1)	23 (56.1)	
High risk	100 (45.7)	55 (44.0)	27 (50.9)	18 (43.9)	
**Equipment**					<0.001
iu22	61	49	12	0	
epiq	148	73	37	38	
other	10	3	4	3	

Continuous variables are summarized as mean ± SD or median [IQR] and compared by one-way ANOVA or Kruskal–Wallis test as appropriate. Categorical variables are compared by chi-square test or Fisher exact test when expected counts are small.

**Table 2:** Selected radiomic features included in the radiomics score formula and their corresponding coefficients.

LASSO Coefficient	Radiomics Features’ Name	Spearman’s Rank Correlation Coefficient
+0.01847	original_shape2D_MajorAxisLength	−0.218541
−2.83658	original_shape2D_PerimeterSurfaceRatio	0.2584433
+3.58671	original_glcm_JointEnergy	−0.04401244
+25.13708	original_glcm_MCC	−0.1446439
+22.90297	original_glrlm_ShortRunLowGrayLevelEmphasis	−0.08531729
+0.10401	original_glszm_GrayLevelVariance	−0.1496336
+0.86997	original_glszm_LowGrayLevelZoneEmphasis	−0.04216334
−0.00033	original_glszm_SizeZoneNonuniformity	−0.1681364
+0.24702	original_glszm_SmallAreaHighGrayLevelEmphasis	0.004105686
+0.39001	original_glszm_ZoneEntropy	−0.1242217
−6.73756	original_glszm_ZonePercentage	0.112624
−0.0078	original_ngtdm_Busyness	−0.2021936
−302.66084	original_ngtdm_Coarseness	0.1905289
+0.06659	original _ngtdm_Complexity	−0.1010771

The radiomics score was derived in the development cohort after LASSO-based feature selection. Positive coefficients increased the radiomics score, whereas negative coefficients decreased the radiomics score.

## Data Availability

The original contributions presented in this study are included in the article/Supplementary Materials. Further inquiries can be directed to the corresponding authors.

## Supplementary Materials

The following supporting information can be downloaded at: https://www.mdpi.com/article/10.3390/curroncol33080464/s1, Table S1: Radiomic features excluded from further analysis because of inter- or intra-observer ICC < 0.80; Table S2: Logistic regression coefficients of the Ki67-rad model for predicting high-risk 70-GS results; Table S3: Diagnostic performance of candidate models in the development, internal validation, and temporally independent validation cohorts; Table S4: Paired DeLong tests comparing the Ki67-radiomics model with combined, clinicopathological, Ki67-only, and radiomics models; Table S5: Bootstrap internal validation and incremental value analysis of the Ki67-rad model and Ki67-only model.

## Abbreviations

The following abbreviations are used in this manuscript:

70-GS	70-gene signature
HR	hormonal receptors
ROI	region of interest
ICC	intraclass correlation coefficient
LASSO	Least Shrinkage and Selection Operator
AUC	area under the receiver operating characteristic curve
HER2	human epidermal growth factor receptor 2
PR	progesterone receptor
ER	estrogen receptor
US	ultrasound
VIF	variance inflation factor
