# Supplementary material for: A Parsimonious Ultrasound Radiomics and Ki-67 Model for Estimating MammaPrint Risk Categorization in HR+/HER2− Early Breast Cancer
Source: Curr Oncol. 2026 Aug 4;33(8):464. doi: 10.3390/curroncol33080464 (PMC13510384; doi:10.3390/curroncol33080464)
Supplement: Supplementary file 1 [file curroncol-33-00464-s001.zip › curroncol-4323557-supplementary.pdf]

Supplementary Table S1. radiomic features excluded from further analysis because of inter- or intra-observer ICC < 0.80

Excluded feature	Intra-observer ICC	Inter-observer ICC
original_firstorder_Minimum	1.000	0.709
original_glcm_ClusterShade	0.916	0.687
original_glcm_ClusterProminence	0.901	0.584
original_ngtdm_Contrast	0.889	0.521
original_glcm_DifferenceVariance	0.869	0.447
original_glcm_Contrast	0.867	0.454
original_glcm_Idmn	0.811	0.441
original_glcm_Correlation	0.725	0.668
original_ngtm_Strength	0.920	0.794
original_firstorder_Skewness	0.704	0.710
original_glcm_Imc2	0.667	0.596
original_firstorder_Kurtosis	0.383	0.401
original_shape2D_Sphericity	0.764	0.860

Supplementary Table S2. Logistic regression coefficients of Ki67-rad model for predicting high-risk 70-GS results.

Predictor	Coding / reference	β coefficient	SE	OR	95% CI	P value
Intercept	—	—	—	—	—	—
Radiomics score	Per 1-unit increase	1.0806	0.2969	2.95	1.71–5.55	<0.001
Ki67	Per 10% increase	0.8109	0.1991	2.25	1.58–3.47	<0.001

Ki67 is presented per 10% increase. 95% confidence intervals are profile-likelihood confidence intervals and p values are Wald test p values.

Supplementary Table S3. Diagnostic performance of candidate models in the development, internal validation, and temporally independent validation cohorts.

Cohort	TP	TN	FN	FP	AUC	SPEC	SEN	PPV	NPV	ACC	OPTIMAL CUTOFF	95%CI of AUC	F1-score
ALL-Tra	40	65	15	5	0.899	0.929	0.727	0.889	0.813	0.84	0.609	0.8451-0.9523	0.8
ALL-Val	23	20	4	6	0.846	0.769	0.852	0.793	0.833	0.811	0.099	0.7362-0.9561	0.821
ALL-Sval	12	20	6	3	0.843	0.87	0.667	0.8	0.769	0.78	0.366	0.7257-0.9603	0.727
CLINIC-Tra	46	50	9	20	0.845	0.714	0.836	0.697	0.847	0.768	0.383	0.7768-0.9136	0.76
CLINIC-Val	20	20	7	6	0.806	0.769	0.741	0.769	0.741	0.755	0.136	0.686-0.9265	0.755
CLINIC-Sval	18	17	0	6	0.915	0.739	1	0.75	1	0.854	0.142	0.8292-1.0000	0.857
RADIOMICS-Tra	39	54	16	16	0.8	0.771	0.709	0.709	0.771	0.744	0.525	0.7222-0.8783	0.709
RADIOMICS-Val	16	19	11	7	0.662	0.731	0.593	0.696	0.633	0.66	0.481	0.5146-0.8102	0.64
RADIOMICS-Sval	8	17	10	6	0.543	0.739	0.444	0.571	0.63	0.61	0.532	0.3558-0.7312	0.5
KI67-RAD-Tra	41	61	14	9	0.878	0.871	0.745	0.82	0.813	0.816	0.554	0.8193-0.9360	0.781
KI67-RAD-Val	23	20	4	6	0.816	0.769	0.852	0.793	0.833	0.811	0.286	0.6946-0.9379	0.821

KI67-RAD-Sval	14	17	4	6	0.831	0.739	0.778	0.7	0.81	0.756	0.393	0.7052-0.9566	0.737
KI67-ONLY-Tra	51	38	4	32	0.826	0.543	0.927	0.614	0.905	0.712	0.297	0.7575-0.8947	0.739
KI67-ONLY-Val	19	23	8	3	0.791	0.885	0.704	0.864	0.742	0.792	0.513	0.6651-0.9175	0.776
KI67-ONLY-Sval	16	16	2	7	0.89	0.696	0.889	0.696	0.889	0.78	0.4	0.7998-0.9804	0.78

Candidate models included the radiomics model, the clinicopathologic model, the Ki67-only model, the Ki67-radiomics model, and the full clinicoradiomic model. AUC, area under the receiver operating characteristic curve; SPEC, specificity; SEN, sensitivity; PPV, positive predictive value; NPV, negative predictive value; ACC, accuracy.

Supplementary Table S4. Paired DeLong tests comparing the Ki67-radiomics model with combined, clinicopathological, Ki67-only, and radiomics models.

Cohort	Comparison	Ki67-radiomics AUC (95% CI)	Compared model AUC (95% CI)	ΔAUC	z	P value
Development cohort	Ki67-rad vs combined model	0.878 (0.819–0.936)	0.897 (0.843–0.952)	-0.020	-1.149	0.251
Development cohort	Ki67-rad vs clinicopathological model	0.878 (0.819–0.936)	0.845 (0.777–0.914)	0.033	1.148	0.251
Development cohort	Ki67-rad vs Ki67-only model	0.878 (0.819–0.936)	0.826 (0.757–0.895)	0.052	2.041	0.041
Development cohort	Ki67-rad vs radiomics model	0.878 (0.819–0.936)	0.801 (0.723–0.879)	0.077	2.588	0.010
Internal validation cohort	Ki67-rad vs combined model	0.816 (0.692–0.940)	0.843 (0.733–0.954)	-0.027	-0.879	0.379
Internal validation cohort	Ki67-rad vs clinicopathological model	0.816 (0.692–0.940)	0.806 (0.686–0.927)	0.010	0.251	0.802
Internal validation cohort	Ki67-rad vs Ki67-only model	0.816 (0.692–0.940)	0.791 (0.663–0.920)	0.025	0.638	0.523
Internal validation cohort	Ki67-rad vs radiomics model	0.816 (0.692–0.940)	0.664 (0.516–0.812)	0.152	2.204	0.028

Temporally independent validation cohort	Ki67-rad vs combined model	0.831 (0.702–0.960)	0.843 (0.717–0.969)	-0.012	-0.252	0.801
Temporally independent validation cohort	Ki67-rad vs clinicopathological model	0.831 (0.702–0.960)	0.906 (0.804–1.000)	-0.075	-1.113	0.266
Temporally independent validation cohort	Ki67-rad vs Ki67-only model	0.831 (0.702–0.960)	0.890 (0.798–0.983)	-0.059	-1.038	0.299
Temporally independent validation cohort	Ki67-rad vs radiomics model	0.831 (0.702–0.960)	0.539 (0.351–0.727)	0.292	3.741	<0.001

AUC, area under the receiver operating characteristic curve. DeLong test is paired at the patient level within each cohort.

Supplementary Table S5. Bootstrap internal validation and incremental value analysis of the Ki67-rad model and Ki67 only model.

Bootstrap internal validation was performed in the development cohort with 1,000 resamples. Reported metrics include apparent AUC, optimism-corrected AUC, corrected calibration slope, and uniform shrinkage factor. Incremental value was assessed by nested likelihood-ratio testing comparing the Ki67-only model with the simplified model including both Ki67 and the radiomics score.

Part A. Bootstrap internal validation in the development cohort.

Model	Apparent AUC	Optimism-corrected AUC	Corrected calibration slope	Uniform shrinkage factor
Ki67-only model	0.826	0.824	0.993	0.993
Ki67-rad model	0.878	0.872	0.953	0.953

Part B. Incremental value of adding the radiomics score to Ki67 in the development cohort.

Comparison	Model 1 log-likelihood	Model 2 log-likelihood	Delta log-likelihood	LR chi-square	df	P value	AIC (Model 1)	AIC (Model 2)
Ki67-only vs Ki67-rad	-63.089	-54.518	8.571	17.143	1	<0.001	130.179	115.036

Abbreviations: AUC, area under the receiver operating characteristic curve; LR, likelihood ratio; AIC, Akaike information criterion. The Ki67-rad model included Ki67 and the radiomics score.
